# Bridging symptoms between problematic social networking and generalized anxiety in adolescents with non-suicidal self-injury: a network analysis

**DOI:** 10.3389/fpsyt.2025.1701263

**Published:** 2026-01-02

**Authors:** Zhen He, Zhige Lyv, Chongchong Li, Jing Lu

**Affiliations:** 1 Department of Preschool Education, Guangxi College for Preschool Education, Nanning, China; 2 College of Education for the Future, Beijing Normal University, Beijing, China; 3 Department of Education, Guilin Normal College, Guilin, China; 4 Guangdong Pharmaceutical University, Guangzhou, China

**Keywords:** non-suicidal self-injury, problematic social networking site use, generalized anxiety, network analysis, adolescence

## Abstract

**Background:**

Problematic social networking site use (PSNSU) may arise as a coping response to generalized anxiety and, in turn, intensify anxiety symptoms, creating a bidirectional cycle that poses increased risks for non-suicidal self-injury (NSSI) among adolescents. However, the symptom-level mechanisms underlying these intertwined associations remain unclear. To address this gap, the present study applied a symptom network analysis approach to examine.

**Methods:**

A total of 5,938 adolescents in Guangxi Province, China, completed an e-questionnaire and validated measures of generalized anxiety, social networking site addictive tendencies, and NSSI. Related to the aim of the current study, 1,544 participants who reported engaging in NSSI were included in the final data analysis, corresponding to a prevalence rate of 26.0% (age *_mean ± sd_*= 18.95 ± 0.90, 22.73% male). Bridge and flow network analyses were estimated using graphical LASSO models with extended Bayesian information criterion selection. Robustness was examined with nonparametric bootstrapping and case-dropping stability (CS-C). Sensitivity analyses controlled for age and gender.

**Results:**

Within the generalized anxiety network, the strongest edge was between uncontrollable worry and excessive worry (edge weight = 0.27). Within the PSNSU network, the most robust edge-linked online relationship satisfaction and virtual friend anxiety (edge weight = 0.35). Cross-community associations indicated irritability was the strongest positive association with social network addiction (edge weight = 0.07). Bridge centrality analysis identified dual existence (*Z* score = 2.47 and above 1) as the key bridge node. Flow network analysis showed that restlessness, irritability, feeling afraid, online relationship satisfaction, and virtual friend anxiety were the only symptoms directly associated with NSSI (edge weight = 0.04, 0.01, 0.03, 0.003, and 0.02, respectively). The network demonstrated strong stability (CS-C = 0.75), and structural consistency was observed after adjusting for covariates, such as age and gender (*r* ≥ 0.99, *p* <.001).

**Conclusions:**

This study reveals that PSNSU, generalized anxiety, and NSSI are interconnected at the symptom level, with “dual existence” being the key bridge linking PSNSU and anxiety, while “restlessness” is the anxiety symptom most directly linked to NSSI. Longitudinal studies are warranted to clarify causal pathways and evaluate symptom-targeted prevention strategies.

## 1 Introduction

With the rapid proliferation of social media, problematic social networking site use (PSNSU) has become an increasingly salient risk factor for mental health, particularly among adolescents (1–3). PSNSU is typically understood as a maladaptive pattern of engagement with social networking sites that leads to functional impairments in daily life. While some scholars conceptualize PSNSU within the framework of behavioral addiction, highlighting features such as impaired control, persistent preoccupation with online interaction, and continued use despite negative consequences (4, 5), others argue that problematic use may also encompass less severe but still detrimental outcomes, such as reduced productivity, sleep disturbance, or relational strain (6, 7). Accordingly, PSNSU can be regarded as a broader construct that overlaps with but is not identical to addictive use.

Several meta-analyses have identified associations between PSNSU and various anxiety-related symptoms, including social anxiety (8), attachment anxiety (9), and fear of missing out (10, 11). Generalized anxiety disorder, regarded as one of the most common mental health concerns among adolescents (12) and young adults (13), has also been repeatedly associated with PSNSU (14–16). Furthermore, frequent engagement with social media platforms may contribute to maladaptive social comparisons (17), intensify negative affective experiences (18), and increase perceived social isolation (19). These factors may collectively elevate the risk of non-suicidal self-injury (NSSI) (20). Despite growing interest in these individual associations, the interconnectedness of PSNSU, generalized anxiety symptoms, and NSSI remains underexplored. This knowledge gap highlights the need for the present investigation to examine their potential interactive effects on adolescents’ and young adults’ psychological well-being.

From a theoretical perspective, compensatory internet use theory (CIUT) provides a better framework to reveal the relation between PSNSU and generalized anxiety symptoms. CIUT posits that PSNSU serves as a compensatory behavior to manage negative emotional states (21). Individuals may turn to social networking sites as a coping mechanism to alleviate psychological distress, particularly anxiety. Empirical evidence suggests that individuals experiencing elevated anxiety levels are more likely to favor computer-mediated communication, as it can provide a heightened sense of social support and fulfil important psychological needs (1), which in turn, may help regulate emotional discomfort (22). However, this reliance is often associated with deficits in emotion regulation (23), which may contribute to the development or persistence of PSNSU (24). Conversely, excessive or maladaptive engagement in social networking sites may further intensify anxiety symptoms through several pathways, such as upward social comparison, fear of negative evaluation, self-control ability, and sleep disruption (25, 26). These mechanisms suggest that the relationship between anxiety and PSNSU is likely to be reciprocal rather than unidirectional. To sum up, anxiety may act as a contributing factor to PSNSU, while PSNSU itself may, in turn, intensify anxiety symptoms or be associated with more severe psychological outcomes, such as NSSI.

NSSI is defined as the intentional infliction of physical harm to one’s own body without suicidal intent, typically employed as a maladaptive strategy for regulating negative emotions, expressing psychological distress, or enacting self-punishment (27). NSSI is particularly prevalent among adolescents and young adults, who are characterized by heightened emotional reactivity and ongoing development of self-regulatory capacities (28). A recent meta-analysis of 264,638 non-clinical adolescents reported a pooled prevalence rate of 22% between 2010 and 2021, indicating a potential upward trend in adolescent engagement in NSSI (29). A growing body of research has consistently demonstrated that NSSI is strongly associated with difficulties in emotion regulation (30), impaired impulse control (31), and a range of adverse social and environmental factors (32). Deficits in emotion regulation represent a transdiagnostic mechanism connecting NSSI, PSNSU, and anxiety. Individuals with poor affect regulation may engage in PSNSU to alleviate distress and seek social reassurance, which can exacerbate anxiety through social comparison, fear of negative evaluation, and sleep disturbance (26). Emotion dysregulation thus provides a conceptual framework for understanding the reciprocal relations among PSNSU, anxiety, and NSSI (30, 33).

Specifically, the affect-regulation model of self-injury (34, 35) suggests that individuals use self-harming behaviors to escape or avoid unpleasant emotional experiences, such as anxiety (36). Empirical research revealed that, among adolescents, NSSI is closely associated with internalizing psychopathology, including anxiety (37), which may worsen social isolation and form negative peer interactions (38), contributing to seeking social support through the use of online social networking sites. A longitudinal study found that problematic smartphone use was associated with NSSI by intensifying symptoms of anxiety and depression over time (39). Other longitudinal studies have reported similar findings, showing that adolescents’ problematic smartphone use or problematic internet use is significantly associated with NSSI (40–43). Moreover, Mészáros et al. (44) noted that while internet use alone may not constitute a direct risk factor for NSSI, it can become one in the presence of comorbid psychiatric conditions, such as generalized anxiety disorder. Although existing studies indicate a link between PSNSU and NSSI (45, 46), the underlying mechanism remains insufficiently understood.

Given that a rich body of research has investigated the relations between PSNSU, generalized anxiety, and NSSI, the precise interconnections among these three mental problems remain insufficiently understood. Furthermore, previous studies have primarily examined the correlations between PSNSU, generalized anxiety, and NSSI based on total scores derived from measurement instruments, which assume homogeneity within each construct and overlook the distinct functional roles of individual symptoms (47). Such variable-centered approaches obscure the complex network of direct and indirect pathways that may sustain comorbidity across these conditions. To address this gap, the present study applies a symptom-level network analysis to map the fine-grained structure of interrelations among PSNSU, anxiety, and NSSI symptoms, identify central and bridge symptoms (i.e., symptoms that are most strongly connected within their own networks or that link distinct symptom clusters across disorders) that may serve as causal maintenance factors, and provide a more mechanistic understanding that traditional correlational methods cannot capture.

Network analysis is an approach that conceptualizes mental disorders as complex networks of interacting symptoms rather than as singular underlying mental disorders (48). By estimating symptom-to-symptom relations, network models can distinguish more direct from indirect pathways, thereby clarifying how specific symptoms influence one another after controlling for the rest. Analyzing the connections between symptoms helps identify central symptoms that are crucial in maintaining or exacerbating mental disorders, offering insights for targeted interventions (49). This methodology has been increasingly applied to comorbidity among digitally related behaviors and internalizing problems, including problematic smartphone use, internet addiction, social anxiety, generalized anxiety, and NSSI (50, 51). Therefore, constructing a symptom-level comorbidity network of generalized anxiety, PSNSU, and NSSI enables us to map putative direct and indirect pathways and isolate candidate causal-maintenance factors (central and bridge symptoms) that variable-centered approaches may obscure. Hence, the current study proposes the following research aims:

Aim 1: To explore the intricate relations among the symptoms of generalized anxiety and PSNSU among adolescents and young adults with NSSI based on symptom network analysis.

Hypothesis 1: Previous studies have highlighted significant associations between PSNSU and generalized anxiety (14–16). Drawing from these findings, we hypothesize that PSNSU and generalized anxiety may form comorbidity networks.

Aim 2: Explore which symptoms within the comorbid anxiety-PSNSU network could be linked to NSSI among adolescents.

Hypothesis 2: Previous studies have established associations between anxiety and NSSI (50, 52, 53). Additionally, research by Elhai et al. (24) suggests that PSNSU may exacerbate generalized anxiety symptoms, with NSSI often functioning as a maladaptive coping mechanism in response to generalized anxiety. Based on these findings, we hypothesize that symptoms of generalized anxiety will act as the most prominent bridges in the network connecting PSNSU symptoms to NSSI among adolescents.

## 2 Methods

### 2.1 Participants

The current study adopted a cross-sectional survey design and employed purposive sampling procedure to recruit 6,395 adolescents and young adults aged 10 to 24 years (54) from four universities in Guangxi Province, China. Data were collected through an online questionnaire platform (https://www.wjx.cn) between May 13 and June 13, 2025.

Before participation, all respondents read an electronic informed consent form. Among them, 149 disagreed with participating after reading the informed consent form. In addition, 308 failed the four attention-check items (i.g, “Please select panda from the following selection. A, tiger. B, monkey. C, panda. D, snake.”). After applying these exclusion criteria, a total of 5,938 participants were retained. Among them, 1,544 participants (prevalence of NSSI is 26%; age *_mean ± sd_*= 18.95 ± 0.90, range from 17 to 23) reported engaging in non-suicidal self-injury (NSSI) based on their endorsement of the item: “I hurt myself intentionally by cutting or burning my skin.” These participants were included in the final data analysis.

The ethics committee of Guilin Normal University reviewed and approved the study protocol on April 30, 2025. For participants under the age of 18, written informed consent was obtained from their legal guardians before they were permitted to complete the survey. The survey was administered anonymously, and participants were allowed to withdraw at any time. Importantly, only participants whose responses were included in the final analysis were debriefed and provided with essential psychological support resources following the survey, to mitigate any potential psychological distress that might have arisen from responding to the NSSI item.

### 2.2 Measurements

#### 2.2.1 Generalized anxiety disorder-7

GAD-7 is a self-report tool for screening generalized anxiety symptoms during the past 2 weeks, which contains seven items (55). Each item was scored from 0 (Not at all) to 3 (Nearly every day). The seven items representing symptoms from the chapter on generalized anxiety disorder of DSM-5 (56), include nervousness, uncontrollable worry, excessive worry, trouble relaxing, restlessness, irritability, and feeling afraid. GAD-7 has been well-validated in Chinese adolescents (57). In the current study, GAD-7 has high internal consistency (*α* = .92).

#### 2.2.2 Short version of social networking sites addictive tendencies scale

Initially, 45 items were created, reflecting the person who may be addicted to social networking sites. In the current study, a brief version consisting of six items was used based on the loading factor and discriminative power (58), as well as being used in the Chinese population (CFI = 0.91, TLI = 0.85, SRMR = 0.05; Cronbach’s *α* = 0.75; 59). All items were five-point Likert type, ranging from one (“Does not describe me at all”) to five (“Describes me completely”). Related to the purpose of the study, each item indicating one symptom that was measuring social networking sites addictive tendencies, include declining productivity, insomnia, dual existence, social network addiction, online relationship satisfaction, and virtual friend anxiety. The current study shows SSNSATS has high internal consistency (*α* = .87).

#### 2.2.3 Non-suicidal self-injury item

The non-suicidal self-injury was measured by one item (“I hurt myself intentionally by cutting or burning my skin.”) (60). Responses were scored on a 4-point, Likert-type scale, where 1 = “never,” 2 = “sometimes,” 3 = “often,” and 4 = “almost. The higher score indicates more severe non-suicidal self-injury.

### 2.3 Statistical procedure

All analyses were conducted in R software (Version 4.5.2; 61).

#### 2.3.1 Estimate bridge and flow network structures

The bridge network was estimated to assess the symptom association between different disorders, which are typically constructed using the graphical Gaussian model (GGM) via the *qgraph* package (62). Specifically, we employed graphical least absolute shrinkage and selection operator (LASSO) network models based on the extended Bayesian information criterion (EBIC) (63) to establish the bridge network structure of social networking site addiction and generalized anxiety symptoms. Specifically, partial correlation analyses were conducted while controlling for all other variables to examine the relationships between each pair of continuous variables (i.e., symptoms) and construct the network structure (64). Within this graphical network, nodes represent individual symptoms, whereas edges denote partial regularized correlations between symptoms. The thickness of the edges indicates the strength of these associations, with blue and red lines signifying positive and negative relations, respectively (65).

Based on the bridge network structure, the flow network was estimated using the qgraph package’s *flow* function (62). This visualization places a focal node on the left and arranges other nodes to the right according to their direct connectivity, forming a tree-like layout. It intuitively illustrates how symptoms are connected to the focal node and helps interpret potential pathways of influence within the network. In the present study, the NSSI symptom was specified as the focal node, followed by the symptoms of PSNSU and generalized anxiety arranged vertically to the right according to their direct connections with NSSI.

#### 2.3.2 Bridge centrality measures

The bridge function in the *networktools* R package (Version 1.4.0) was employed to identify bridge symptoms within the network connecting two or more psychiatric disorders (66). Following the methodology of a previous study (67), bridge symptoms were determined using the bridge expected influence (1-step) (BEI) within the network, considering both positive and negative relationships. According to previous studies (68, 69), the *Z* score of node’s BEI above 1 can be a key bridge symptom linking social networking site addiction and generalized anxiety symptoms.

#### 2.3.3 Network stability and accuracy

The *bootnet* (version 1.5.6) package (70) was used to examine the bridge network architecture’s robustness.

Firstly, network stability was assessed via a case-reduction bootstrap procedure to compute the correlation stability coefficient (CS-C). This metric specifies the maximal proportion of cases that may be excluded whilst retaining a Pearson’s correlation ≥ 0.7 (95% *CI*) between the original BEI centrality measures and those estimated from subsampled networks. In accordance with methodological guidelines (70), a CS-C value ≥ 0.25 was considered minimally acceptable, though a value exceeding 0.5 reflects preferable stability. Secondly, the nonparametric bootstrapping (1,000 iterations, with replacement) was employed to quantify edge-weight precision, with 95% confidence intervals derived from the resultant sampling distributions. Bootstrap difference testing was then conducted to evaluate statistical discrepancies in network parameters, including pairwise edge weights and BEI indices. Finally, nodal predictability, which is operationalized as the variance proportion in each node explained by its topological neighbors, was estimated using the *mgm* (version 1.2-14) package (71). This measure provides insight into the extent to which local network connections account for individual node variability.

#### 2.3.4 Sensitivity analysis

To account for the influence of gender and age on network estimation, the current study incorporated gender and age as covariates in the network analysis (72, 73). Then, network matrices were compared with those derived from analyses excluding these covariates, and a high similarity between the two matrices indicated that gender and age did not meaningfully affect the estimated network structure.

## 3 Results

### 3.1 Descriptive statistics

The means and standard deviations of GAD-7, SSNSATS, and NSSI items are shown in **Table 1**. Meanwhile, the basic information of participants shows that 351 (22.73%) males participated in the present study, and the mean age of all participants was 18.95 years.

**Table 1:** Bias information and descriptive item analysis.

Variables	Abbreviation	*Mean*	Percentage or *SD*	Predictability
Gender				
Male		351	22.73%	
Female		1193	77.27%	
Age		18.95	0.9	
GAD1	Nervousness	0.46	0.61	0.58
GAD2	Uncontrollable worry	0.35	0.59	0.68
GAD3	Excessive worry	0.47	0.65	0.66
GAD4	Trouble relaxing	0.37	0.6	0.62
GAD5	Restlessness	0.23	0.49	0.56
GAD6	Irritability	0.39	0.59	0.53
GAD7	Feeling afraid	0.26	0.51	0.53
SNS1	Declining productivity	2.06	1.07	0.46
SNS2	Insomnia	2.32	1.24	0.47
SNS3	Dual existence	2.34	1.29	0.56
SNS4	Social network addiction	2.01	1.11	0.55
SNS5	Online relationship satisfaction	1.95	1.1	0.46
SNS6	Virtual friend anxiety	1.91	1.04	0.45
NSSI	Non-suicidal self-injury	2.04	0.28	0.02

Predictability means R-squared (i.e., the variance proportion in each node explained by its topological neighbors).

### 3.2 Bridge network structure and centrality measures

**Figure 1:**
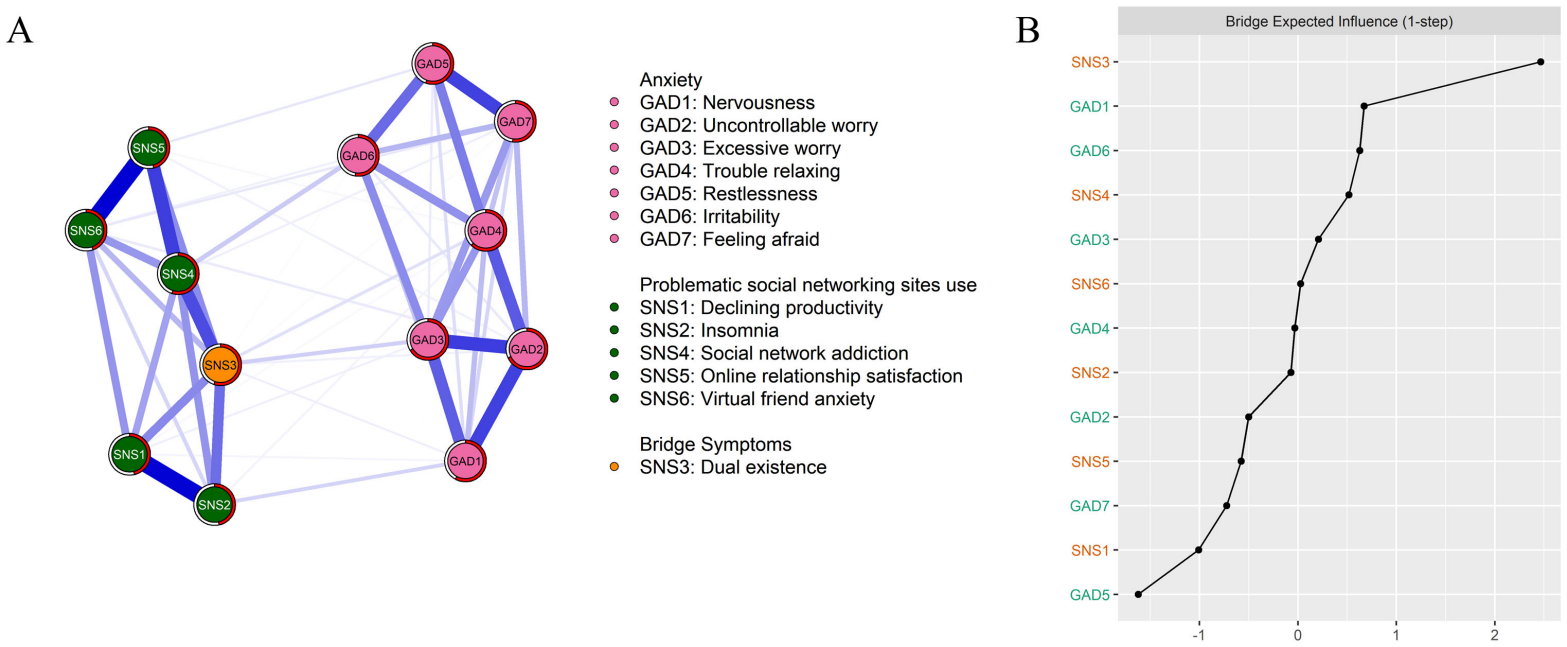
Bridge network structure and bridge centrality index. **(A)** bridge network structure. **(B)** standard bridge centrality index of the bridge network structure.

The structure of the PSNSU and generalized anxiety symptom network is presented in **Figure 1** and **Supplementary Table S1**. Several findings merit attention.

As shown in Part A of **Figure 1**, the strongest edge within the anxiety network was observed between GAD2 (uncontrollable worry) and GAD3 (excessive worry; edge weight = 0.27), whereas in the PSNSU network, the strongest edge emerged between SNS5 (online relationship satisfaction) and SNS6 (virtual friend anxiety; edge weight = 0.35). While across the anxiety-PSNSU network, the strongest edge was GAD6 (irritability) and SNS4 (social network addiction; edge weight = 0.07).

Regarding bridge centrality, SNS3 (dual existence) emerged as key bridge nodes, with *Z* scores above 1, thereby linking PSNSU and generalized anxiety symptoms (see Part B of **Figure 1**).

### 3.3 Network structure robustness

**Figure 2:**
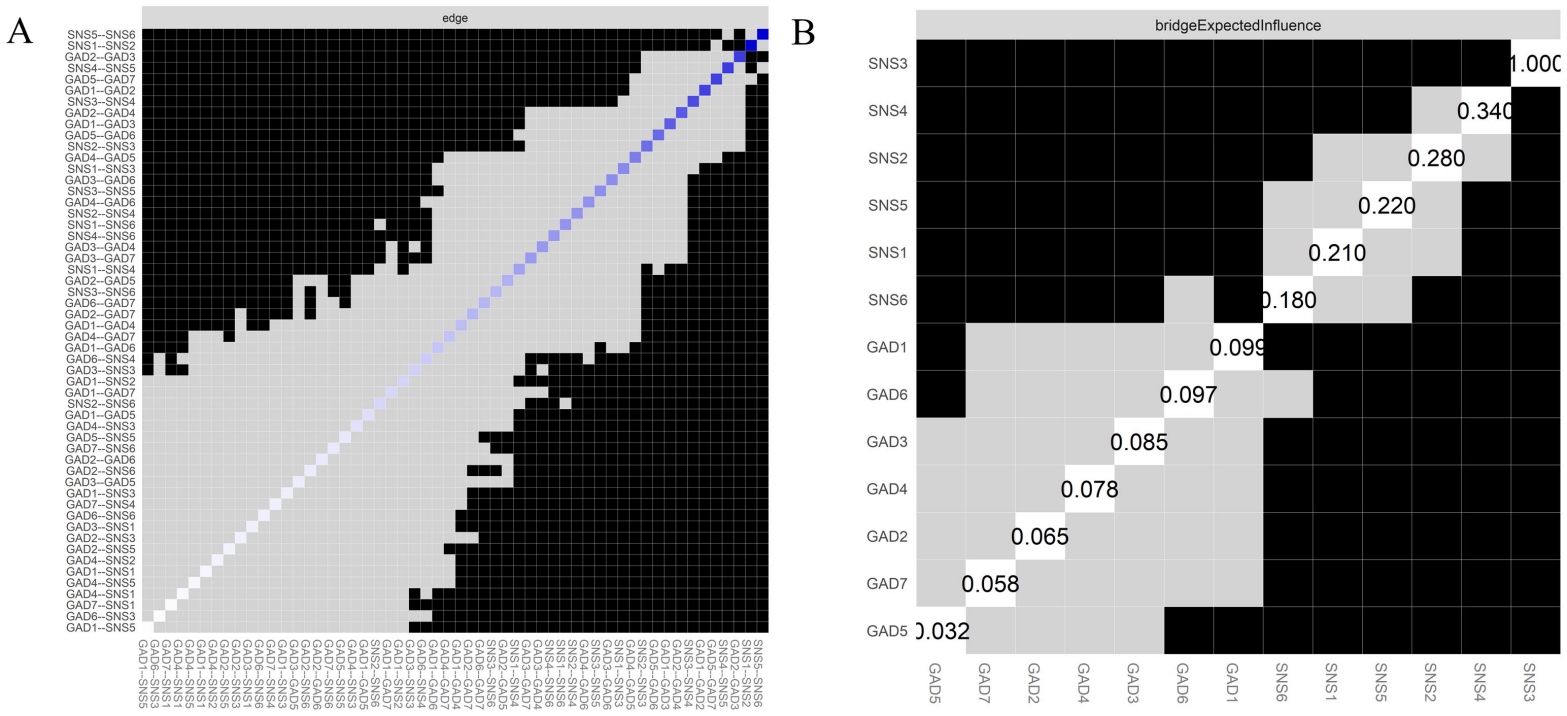
Bootstrapped accurately test for edge-weights and bridge EI values. **(A)** the color of the boxes indicates whether edge-weights differ significantly from each other (i.e., black) or do not differ significantly (i.e., grey). The diagonal line indicates the strength of edge-weights, shifting from red (negative associations) to white (representing weaker edges) and ultimately blue (representing stronger edge-weights). **(B)** grey boxes indicate no significant difference, whereas black boxes indicate a statistically significant difference (*p* < 0.05).

In **Supplementary Figure S1**, the narrow 95% confidence intervals (*CI*s) obtained through the bootstrapping procedure provide evidence that the network edges are robust and reliable. The nonparametric bootstrapping analysis revealed that the edge weights of SNS5 (online relationship satisfaction) - SNS6 (virtual friend anxiety) and GAD2 (uncontrollable worry) - GAD3 (excessive worry) were significantly different from other edge weights, as shown in part A of **Figure 2**. Additionally, SNS3 (Dual existence) was statistically significant compared to the remaining symptoms, indicating that dual existence was the most accurately identified symptom (see part B of **Figure 2**).

Furthermore, the case-dropping bootstrapping analysis results showed that the *CS-C* values were 0.75, demonstrating the stability of the BEI values (see **Supplementary Figure S2**).

### 3.4 Flow network structure

**Figure 3:**
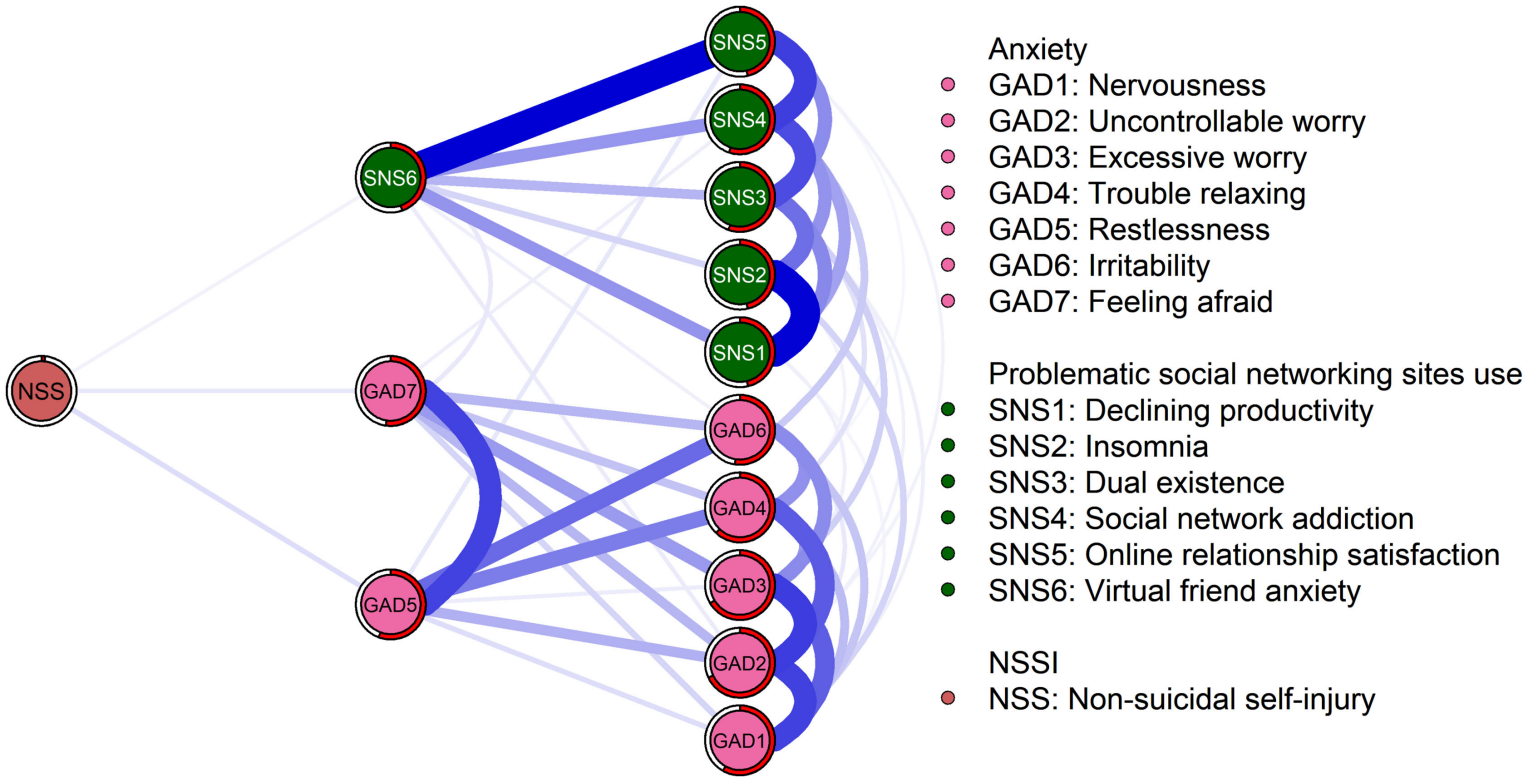
Flow network.

**Supplementary Table S2** and **Figure 3** illustrate the comorbidity network between PSNSU and generalized anxiety symptoms related to NSSI. Among the 13 symptoms examined, GAD5 (restlessness), GAD6 (irritability), GAD7 (feeling afraid), SNS5 (online relationship satisfaction), and SNS6 (virtual friend anxiety) were directly linked to NSSI.

### 3.5 Sensitivity analysis

Sensitivity analysis results demonstrate a significant correlation between the bridge network structure without covariates and the bridge network structure with covariates (*r* = 0.99, *p* <.001). Similarly, the flow network structure without covariates was significantly correlated with the flow network structure with covariates (*r* = 0.99, *p* <.001). These findings suggest that the current network structure is not influenced by gender or age.

## 4 Discussion

This study investigates the interrelations between PSNSU and generalized anxiety symptoms and their impact on NSSI by employing symptom networks and a flow network analysis. We also conducted robustness checks, including the nonparametric bootstrapping of edge-weights and centrality bridge symptoms, case-dropping stability, and covariate-adjusted networks, which together indicate that the observed topology is stable. Some interesting findings are worth discussing.

First, within the anxiety symptom network, the edge weight between uncontrollable worry and excessive worry was the strongest, indicating a close association between these two generalized anxiety symptoms. Excessive worry, defined as persistent and disproportionate concerns across various domains of life (55), may lead to a heightened sense of uncontrollability (57), particularly among adolescents who are in the process of developing executive functioning and emotion regulation skills (74). What’s more, the strongest edge found in the current study is consistent with existing research on generalized anxiety, where uncontrollable worry is considered a central symptom among adolescents (75). From a network perspective, the strong interconnection indicates an interrelation association whereby excessive worry intensifies the perceived lack of control over thoughts, and conversely, the perceived lack of control exacerbates excessive worry. This mutually reinforcing dyad helps explain the persistence of worry in youth and may be compounded by individual differences in emotion regulation capacities (76) and personality traits (77).

Within the PSNSU network, the strongest association emerged between online relationship satisfaction and virtual friend anxiety, indicating that while individuals may derive a sense of fulfillment and belonging from online interactions, they simultaneously experience heightened anxiety about sustaining these virtual connections (78). This dual experience reflects a paradoxical dynamic in which online social gratifications coexist with fears of rejection, loss of reciprocity, or perceived inadequacy in digital relationships, reinforcing compulsive engagement with social networking sites. Excessive social networking site engagement, especially at night, disrupts circadian rhythms and sleep hygiene and may lead to insomnia, which, in turn, impairs cognitive performance (78), motivation (79), and attention during the day (80), reducing academic and functional productivity (81). Additionally, adolescents may use social networking sites as a coping strategy for stress, reinforcing nighttime usage and sleep delay. For example, previous studies have highlighted that excessive use of social networking sites disrupts sleep, which in turn exacerbates symptoms of anxiety and depressive disorders (82). The association between declining productivity and insomnia is a crucial insight, suggesting that problematic social networking site use (rather than assuming clinical addiction) is not only detrimental to mental health but also impairs occupational and academic performance, which can perpetuate anxiety and stress (7, 83). Hence, we can infer that insomnia and daytime impairment likely form a short feedback loop, which means that sleep loss undermines productivity, and reduced productivity increases stress and compensatory late-night use, thereby further delaying sleep.

Interestingly, the PSNSU symptom dual existence (i.e., a perceived split between one’s online and offline identities, where individuals maintain an idealized digital self that diverges from their real-life self, leading to cognitive dissonance and anxiety) emerged as a particularly influential bridge connecting the two symptom domains, consistent with our hypothesis 1. This suggests that adolescents who feel they are leading a “dual life” online versus offline may experience heightened generalized anxiety that intertwines with their SNS use behaviors. These findings align with prior research suggesting that anxiety and PSNSU often co-occur, creating a feedback loop that worsens each other (84). Dual existence served as a significant bridge symptom, which is supported by previous research, which indicates that the phenomenon whereby individuals cannot distinguish between reality and virtuality often mediates the relationship between anxiety and addictive behaviors (23, 33, 84, 85).

In addition to this bridging role, the strongest cross-community edge in the network was observed between GAD6 (irritability) and SNS4 (social network addiction). This direct connection implies a distinct emotional and behavioral cycle in which irritability may drive compulsive social network use as an attempt to discharge or distract from negative affect, while excessive use in turn amplifies irritability through frustration, social overload, or unmet online expectations (86). Clinically, addressing irritability as an affective trigger for compulsive online engagement through emotion regulation and impulse control strategies may help interrupt this cycle and reduce vulnerability to PSNSU-related anxiety. The dual role of social network addiction and irritability in linking PSNSU with generalized anxiety suggests that interventions targeting these symptoms might be effective in breaking the cycle of PSNSU and generalized anxiety, providing a potential therapeutic target for clinical practice.

The present study also explored the relation between PSNSU, generalized anxiety symptoms, and NSSI. Consistent with the flow network and our hypothesis 2, restlessness was the strongest symptom directly linked to NSSI. This finding suggests that the hyperarousal or psychomotor agitation characteristic of generalized anxiety may heighten vulnerability to NSSI as a maladaptive attempt to down-regulate intolerable arousal states, consistent with affect-regulation models of self-injury (34, 35). Previous studies have emphasized the connection between emotional dysregulation and NSSI (30), particularly among individuals with anxiety disorders (50). Moreover, the current results suggest that NSSI was most closely associated with the interplay between PSNSU and anxiety symptoms, rather than a unidirectional pathway from PSNSU to NSSI. This interpretation is supported by the network pattern, in which PSNSU and anxiety nodes showed high interconnectivity and jointly bridged to NSSI, implying a mutually reinforcing process where excessive online engagement and anxious hyperarousal co-escalate, increasing self-injury risk. Clinically, these findings highlight the importance of interventions that concurrently target physiological arousal (e.g., restlessness reduction through relaxation or grounding techniques) and maladaptive digital coping behaviors, thereby mitigating NSSI risk among adolescents experiencing problematic SNS use and anxiety.

### 4.1 Limitations

Some limitations should be noted. First, the cross-sectional study design does not support the causal relations between PSNSU, generalized anxiety symptoms, and NSSI. Hence, the interpretation of pathways should be cautious and regarded as hypothesis−generating. Second, although the current study identified restlessness as the anxiety symptom most proximally linked to NSSI in the flow network, there is a lack of intervention studies to validate this role. Third, the analytic sample used for network estimation comprised only participants who endorsed NSSI, which may limit generalizability to adolescents without recent self−injury and could inflate associations proximal to NSSI. Fourth, basic sociodemographic characteristics such as educational level, residence (rural/urban), and family economic status were not collected, which may further constrain the generalizability of the findings. Fifth, all variables were assessed via self-report, introducing common-method bias; and ordinal items (e.g., Likert scales) were modeled as continuous in the GGM, which may affect edge estimation. Sixth, NSSI was measured with a single item focused on cutting/burning, potentially underrepresenting other common NSSI methods (e.g., scratching, hitting) and functions; future work should adopt multi−item, multi−method assessments (87). Finally, the SSNSATS captures “addictive tendencies” and some consequence−focused items (e.g., productivity decline), which may partially overlap conceptually with outcomes in the network (e.g., insomnia), warranting cautious interpretation of cross−domain edges.

## 5 Conclusions

The current study provides a comprehensive analysis of the interplay between PSNSU, generalized anxiety symptoms, and NSSI. The results suggest that the PSNSU symptom “dual existence” links generalized anxiety symptoms and PSNSU. Furthermore, generalized anxiety symptoms appear to NSSI directly than PSNSU symptoms in the flow network. These findings underscore the importance of integrated clinical approaches that simultaneously target dual existence through emotion regulation training interventions while also reducing high-risk social media patterns such as late-night usage. Future research should investigate the causal mechanisms linking PSNSU, generalized anxiety symptoms, and NSSI and develop targeted interventions that can interrupt the reciprocal cycles among generalized anxiety, PSNSU, and NSSI.

## Data Availability

The raw data supporting the conclusions of this article will be made available by the authors, without undue reservation.
