# Supplementary material for: Bridging symptoms between problematic social networking and generalized anxiety in adolescents with non-suicidal self-injury: a network analysis
Source: Front Psychiatry. 2026 Jan 2;16:1701263. doi: 10.3389/fpsyt.2025.1701263 (PMC12808407; doi:10.3389/fpsyt.2025.1701263)
Supplement: Supplementary file 2 [file Table2.docx]

#########################

rm(list = ls())

#### install and load packages ###############

packages = c("qgraph", "dplyr","networktools", "mgm","PerformanceAnalytics",

"psych","formattable","gt","gtExtras","gtsummary","formattable",

"NetworkComparisonTest","bootnet","apaTables","effectsize",

"IsingSampler","nodeIdentifyR","tidyr","purrr","ggpubr",

"stargazer","ggplot2","readxl","compareGroups","effsize")

package.check <- lapply(

packages,

FUN = function(x) {

if (!require(x, character.only = TRUE)) {

install.packages(x, dependencies = TRUE)

library(x, character.only = TRUE) }})

########## pre-coding

In_Data <- read_excel("In_Data.xlsx")

In_Data1 <- subset(In_Data,Agree==1)########### delete participants who disagree

In_Data2 <- subset(In_Data1,WEITI1==4)########## attention-check

In_Data2$NSSI_Group <- ifelse(In_Data2$NSSI==1,0,1) ############## NSSI group

In_Data2$NSSI_Group <- factor(In_Data2$NSSI_Group,levels = c("0","1"),

labels=c("Non-NSSI","NSSI"))

In_Data2$Gender <- factor(In_Data2$Gender,levels = c("1","2"),

labels=c("Male","Female"))

##########################################

Final_Data <- subset(In_Data2,NSSI_Group=="NSSI")#### 1544 participants

DT <- descrTable( ~ Gender+Age+GAD1+GAD2+GAD3+GAD4+GAD5+GAD6+GAD7+SNS1+SNS2+SNS3+SNS4+

SNS5+SNS6+NSSI,Final_Data, digits = 2)

export2xls(DT,file = "DT.xlsx")

######### legends

legend1 <- c(

"Nervousness",

"Uncontrollable worry",

"Excessive worry",

"Trouble relaxing",

"Restlessness",

"Irritability",

"Feeling afraid",

"Declining productivity",

"Insomnia",

"Dual existence",

"Social network addiction",

"Online relationship satisfaction",

"Virtual friend anxiety")

legend2 <- c(

"Nervousness",

"Uncontrollable worry",

"Excessive worry",

"Trouble relaxing",

"Restlessness",

"Irritability",

"Feeling afraid",

"Declining productivity",

"Insomnia",

"Dual existence",

"Social network addiction",

"Online relationship satisfaction",

"Virtual friend anxiety",

"Non-suicidal self-injury")

##### bridge network

set.seed(123)

Net_Bridge <- estimateNetwork(Final_Data[,c(2:8,12:17)],default = "EBICglasso",corMethod = "spearman",threshold=FALSE)

########### flow network

set.seed(123)

Net_Flow <- estimateNetwork(Final_Data[,c(2:8,12:17,18)],default = "EBICglasso",corMethod = "spearman",threshold=FALSE)

########################

AD_Bridge <- Net_Bridge$graph %>% as.data.frame()

writexl::write_xlsx(AD_Bridge,path = "AD_Bridge.xlsx")

AD_Net_Flow <- Net_Flow$graph %>% as.data.frame()

writexl::write_xlsx(AD_Net_Flow,path = "AD_Net_Flow.xlsx")

############# bridge caulate

Bridge_Net<- bridge(Net_Bridge$graph,list(Anxiety=1:7,SNS=8:13))

tiff("Bridge_Net.tiff",width = 2200, height = 2800,units = 'px',res=500)

plot(Bridge_Net, order="value", zscore=TRUE,color = TRUE,

include=c("Bridge Expected Influence (1-step)"))

dev.off()

#### R square for Bridge_Net

MGM_Net_Bridge <- as.matrix(Final_Data[,c(2:8,12:17)])

set.seed(123)

Fit_Net_Bridge <- mgm(data = MGM_Net_Bridge,type =rep('g',13),

lambdaSel = 'CV',ruleReg = 'OR', pbar = FALSE )

Pred_Net_Bridge <- predict(object = Fit_Net_Bridge,data=MGM_Net_Bridge)

R2_Net_Bridge <- c(Pred_Net_Bridge$error[,3])

write.table(R2_Net_Bridge, file="R2_Net_Bridge.csv", sep = ",",col.names = FALSE)

mean(R2_Net_Bridge)#0.55

sd(R2_Net_Bridge)# 0.08

#### R square for Net_Flow

MGM_Net_Flow <- as.matrix(Final_Data[,c(2:8,12:17,18)])

set.seed(123)

Fit_Net_Flow <- mgm(data = MGM_Net_Flow,type =rep('g',14),

lambdaSel = 'CV',ruleReg = 'OR', pbar = FALSE )

Pred_Net_Flow <- predict(object = Fit_Net_Flow,data=MGM_Net_Flow)

R2_Net_Flow <- c(Pred_Net_Flow$error[,3])

write.table(R2_Net_Flow, file="R2_Net_Flow.csv", sep = ",",col.names = FALSE)

mean(R2_Net_Flow)#0.51

sd(R2_Net_Flow)# 0.16

#######

groups_Net_Bridge=list("Anxiety"=c(1:7),

"Problematic social networking sites use"=c(8:9,11:13),

"Bridge Symptoms"=c(10))

qgraph(Net_Bridge$graph,layout="spring",pie=Pred_Net_Bridge$error[,3],

pieColor="red", nodeNames = legend1, legend.cex=0.7,

shape="circle",

filetype="tiff",filename="Net_Bridge",

legend.mode="style1",GLratio=1.2,node.width=0.6,node.height=0.6,

theme="colorblind", color=c("#EE6AA7", "#006400","#FF8C00"),

groups=groups_Net_Bridge)

#######

groups_Net_Flow=list("Anxiety"=1:7,

"Problematic social networking sites use"=8:13,

"NSSI"=14)

Net_Flow_Qgraph <- qgraph(Net_Flow$graph,layout="spring",pie=Pred_Net_Flow$error[,3],

pieColor="red", nodeNames = legend2, legend.cex=0.7,

shape="circle",

filetype="tiff",filename="Flow",threshold=0.01,

legend.mode="style1",GLratio=1,node.width=0.6,node.height=0.6,

theme="colorblind", color=c("#EE6AA7", "#006400", "#CD5C5C"),

groups=groups_Net_Flow)

flow(Net_Flow_Qgraph,14,horizontal = TRUE)

################################

################### edge stability

set.seed(123)

Bootnet_Bridge <- bootnet(Net_Bridge, nBoots = 1000, nCores = 16,communities=groups_Net_Bridge,

statistics = c("edge", "bridgeExpectedInfluence"))

save(Bootnet_Bridge,file = "Bootnet_Bridge.Rdata")

#########

tiff("Bootnet_Bridge1.tiff",width = 4800, height = 4800,units = 'px',res=600,

compression = "lzw")

plot(Bootnet_Bridge, order = "sample",labels = TRUE) ## egde bootstrap

dev.off()

##########

tiff("Bootnet_Bridge2.tiff",width = 3800, height = 3800,units = 'px',res=600,

compression = "lzw")

plot(Bootnet_Bridge, "edge", plot = "difference",onlyNonZero = TRUE,labels = TRUE,

order = "sample")

dev.off()

###########

tiff("Bootnet_Bridge3.tiff",width = 2900, height = 2900,units = 'px',res=600,

compression = "lzw")

plot(Bootnet_Bridge, "bridgeExpectedInfluence", plot = "difference",labels = TRUE,order="sample",

alpha=0.05)

dev.off()

################## drop-case bootstrap

set.seed(123)

Results_Bridge <- bootnet(Net_Bridge, nBoots = 1000, nCores = 8,communities=groups_Net_Bridge,

statistics = c("bridgeExpectedInfluence"),

type = "case")

save(Results_Bridge,file = "Results_Bridge.Rdata")

# Plot centrality stability:

tiff("Results_Bridge.tiff",width = 2800, height = 2800,units = 'px',res=600,

compression = "lzw")

plot(Results_Bridge,statistics = c("bridgeExpectedInfluence"))

dev.off()

# Compute CS-coefficients:

corStability(Results_Bridge)

###################################

##trans effect

In_Data <- read_excel("In_Data.xlsx")

In_Data1 <- subset(In_Data,Agree==1)########### delete participants who disagree

In_Data2 <- subset(In_Data1,WEITI1==4)########## attention-check

In_Data2$NSSI_Group <- ifelse(In_Data2$NSSI==1,0,1) ############## NSSI group

In_Data2$NSSI_Group <- factor(In_Data2$NSSI_Group,levels = c("0","1"),

labels=c("Non-NSSI","NSSI"))

Final_Data <- subset(In_Data2,NSSI_Group=="NSSI")#### 1544 participants

########### Sensitivity network for bridge and flow network

#####################

set.seed(123)

Net_Bridge2 <- estimateNetwork(Final_Data[,c(2:10,12:17)],default = "EBICglasso",corMethod = "spearman",threshold=FALSE)

########### flow network

set.seed(123)

Net_Flow2 <- estimateNetwork(Final_Data[,c(2:10,12:17,18)],default = "EBICglasso",corMethod = "spearman",threshold=FALSE)

library(vegan)

mantel(Net_Bridge$graph,Net_Bridge2$graph[c(1:7,10:15),c(1:7,10:15)],method = 'spearman')

mantel(Net_Flow$graph,Net_Flow2$graph[c(1:7,10:16),c(1:7,10:16)],method = 'spearman')
