# Supplementary material for: Bridging symptoms between problematic social networking and generalized anxiety in adolescents with non-suicidal self-injury: a network analysis
Source: Front Psychiatry. 2026 Jan 2;16:1701263. doi: 10.3389/fpsyt.2025.1701263 (PMC12808407; doi:10.3389/fpsyt.2025.1701263)
Supplement: Supplementary file 3 [file Supplementaryfile1.docx]

Table S1. The edge weight of the bridge network structure.

|  | GAD1 | GAD2 | GAD3 | GAD4 | GAD5 | GAD6 | GAD7 | SNS1 | SNS2 | SNS3 | SNS4 | SNS5 | SNS6 |
| --- | --- | --- | --- | --- | --- | --- | --- | --- | --- | --- | --- | --- | --- |
| GAD1 | 0.00 | 0.26 | 0.22 | 0.09 | 0.05 | 0.08 | 0.06 | 0.01 | 0.06 | 0.02 | 0.00 | 0.00 | 0.00 |
| GAD2 | 0.26 | 0.00 | 0.27 | 0.23 | 0.11 | 0.03 | 0.10 | 0.00 | 0.00 | 0.02 | 0.00 | 0.02 | 0.03 |
| GAD3 | 0.22 | 0.27 | 0.00 | 0.14 | 0.03 | 0.16 | 0.14 | 0.02 | 0.00 | 0.06 | 0.00 | 0.00 | 0.00 |
| GAD4 | 0.09 | 0.23 | 0.14 | 0.00 | 0.18 | 0.15 | 0.08 | 0.01 | 0.02 | 0.04 | 0.00 | 0.01 | 0.00 |
| GAD5 | 0.05 | 0.11 | 0.03 | 0.18 | 0.00 | 0.21 | 0.26 | 0.00 | 0.00 | 0.00 | 0.00 | 0.03 | 0.00 |
| GAD6 | 0.08 | 0.03 | 0.16 | 0.15 | 0.21 | 0.00 | 0.10 | 0.00 | 0.00 | 0.00 | 0.07 | 0.00 | 0.02 |
| GAD7 | 0.06 | 0.10 | 0.14 | 0.08 | 0.26 | 0.10 | 0.00 | 0.01 | 0.00 | 0.00 | 0.02 | 0.00 | 0.03 |
| SNS1 | 0.01 | 0.00 | 0.02 | 0.01 | 0.00 | 0.00 | 0.01 | 0.00 | 0.35 | 0.16 | 0.13 | 0.00 | 0.15 |
| SNS2 | 0.06 | 0.00 | 0.00 | 0.02 | 0.00 | 0.00 | 0.00 | 0.35 | 0.00 | 0.20 | 0.15 | 0.00 | 0.06 |
| SNS3 | 0.02 | 0.02 | 0.06 | 0.04 | 0.00 | 0.00 | 0.00 | 0.16 | 0.20 | 0.00 | 0.25 | 0.16 | 0.10 |
| SNS4 | 0.00 | 0.00 | 0.00 | 0.00 | 0.00 | 0.07 | 0.02 | 0.13 | 0.15 | 0.25 | 0.00 | 0.27 | 0.14 |
| SNS5 | 0.00 | 0.02 | 0.00 | 0.01 | 0.03 | 0.00 | 0.00 | 0.00 | 0.00 | 0.16 | 0.27 | 0.00 | 0.35 |
| SNS6 | 0.00 | 0.03 | 0.00 | 0.00 | 0.00 | 0.02 | 0.03 | 0.15 | 0.06 | 0.10 | 0.14 | 0.35 | 0.00 |

Table S2. The edge weight of the flow network structure.

|  | GAD1 | GAD2 | GAD3 | GAD4 | GAD5 | GAD6 | GAD7 | SNS1 | SNS2 | SNS3 | SNS4 | SNS5 | SNS6 | ISSN |
| --- | --- | --- | --- | --- | --- | --- | --- | --- | --- | --- | --- | --- | --- | --- |
| GAD1 | 0.00 | 0.26 | 0.22 | 0.09 | 0.05 | 0.08 | 0.06 | 0.01 | 0.06 | 0.02 | 0.00 | 0.00 | 0.00 | 0.00 |
| GAD2 | 0.26 | 0.00 | 0.27 | 0.23 | 0.11 | 0.03 | 0.10 | 0.00 | 0.00 | 0.02 | 0.00 | 0.02 | 0.03 | 0.00 |
| GAD3 | 0.22 | 0.27 | 0.00 | 0.14 | 0.03 | 0.16 | 0.14 | 0.02 | 0.00 | 0.06 | 0.00 | 0.00 | 0.00 | 0.00 |
| GAD4 | 0.09 | 0.23 | 0.14 | 0.00 | 0.18 | 0.15 | 0.08 | 0.01 | 0.02 | 0.04 | 0.00 | 0.01 | 0.00 | 0.00 |
| GAD5 | 0.05 | 0.11 | 0.03 | 0.18 | 0.00 | 0.21 | 0.26 | 0.00 | 0.00 | 0.00 | 0.00 | 0.03 | 0.00 | 0.05 |
| GAD6 | 0.08 | 0.03 | 0.16 | 0.15 | 0.21 | 0.00 | 0.10 | 0.00 | 0.00 | 0.00 | 0.07 | 0.00 | 0.02 | 0.01 |
| GAD7 | 0.06 | 0.10 | 0.14 | 0.08 | 0.26 | 0.10 | 0.00 | 0.01 | 0.00 | 0.00 | 0.02 | 0.00 | 0.03 | 0.03 |
| SNS1 | 0.01 | 0.00 | 0.02 | 0.01 | 0.00 | 0.00 | 0.01 | 0.00 | 0.35 | 0.16 | 0.13 | 0.00 | 0.15 | 0.00 |
| SNS2 | 0.06 | 0.00 | 0.00 | 0.02 | 0.00 | 0.00 | 0.00 | 0.35 | 0.00 | 0.20 | 0.15 | 0.00 | 0.06 | 0.00 |
| SNS3 | 0.02 | 0.02 | 0.06 | 0.04 | 0.00 | 0.00 | 0.00 | 0.16 | 0.20 | 0.00 | 0.25 | 0.16 | 0.10 | 0.00 |
| SNS4 | 0.00 | 0.00 | 0.00 | 0.00 | 0.00 | 0.07 | 0.02 | 0.13 | 0.15 | 0.25 | 0.00 | 0.27 | 0.14 | 0.00 |
| SNS5 | 0.00 | 0.02 | 0.00 | 0.01 | 0.03 | 0.00 | 0.00 | 0.00 | 0.00 | 0.16 | 0.27 | 0.00 | 0.35 | 0.00 |
| SNS6 | 0.00 | 0.03 | 0.00 | 0.00 | 0.00 | 0.02 | 0.03 | 0.15 | 0.06 | 0.10 | 0.14 | 0.35 | 0.00 | 0.02 |
| ISSN | 0.00 | 0.00 | 0.00 | 0.00 | 0.05 | 0.01 | 0.03 | 0.00 | 0.00 | 0.00 | 0.00 | 0.00 | 0.02 | 0.00 |


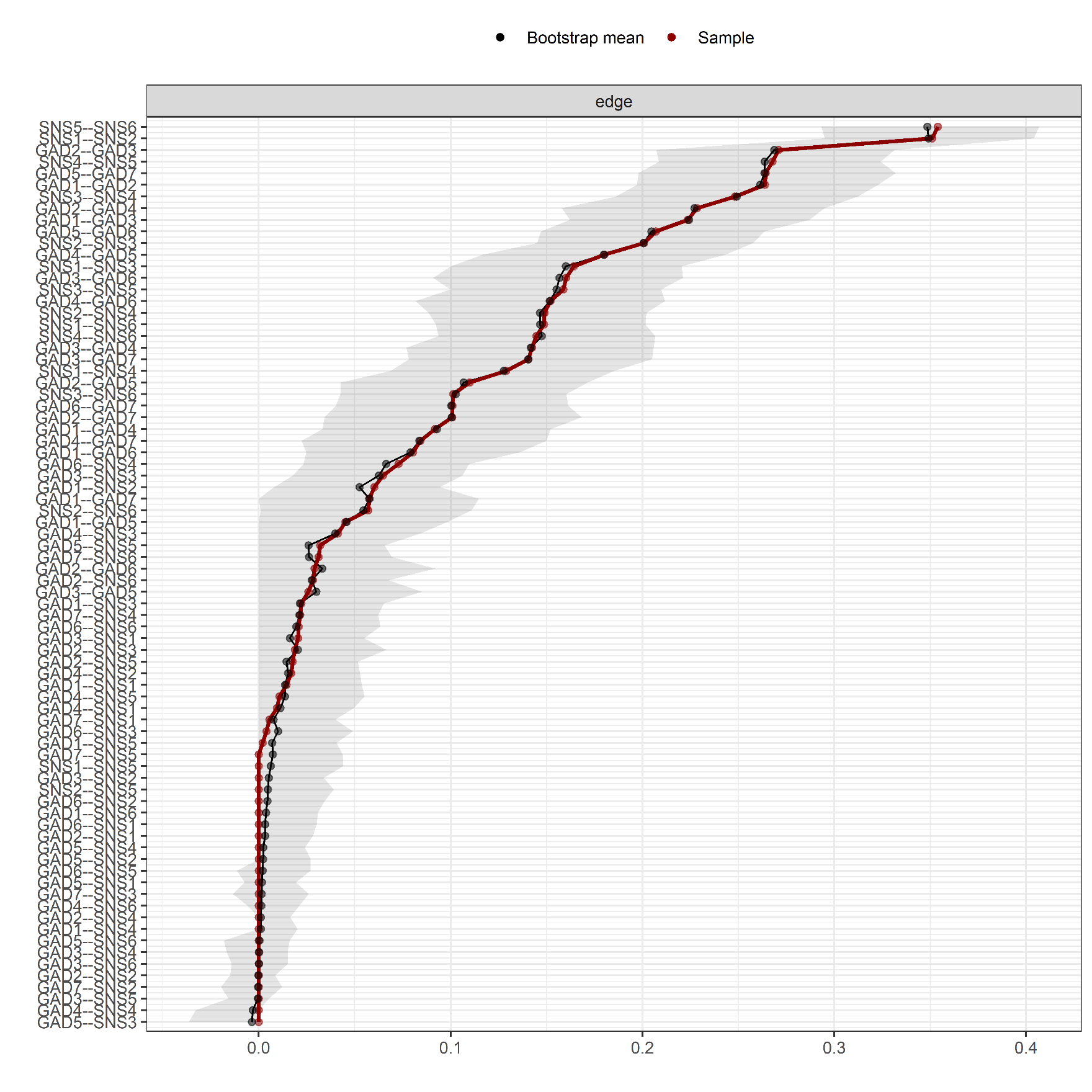


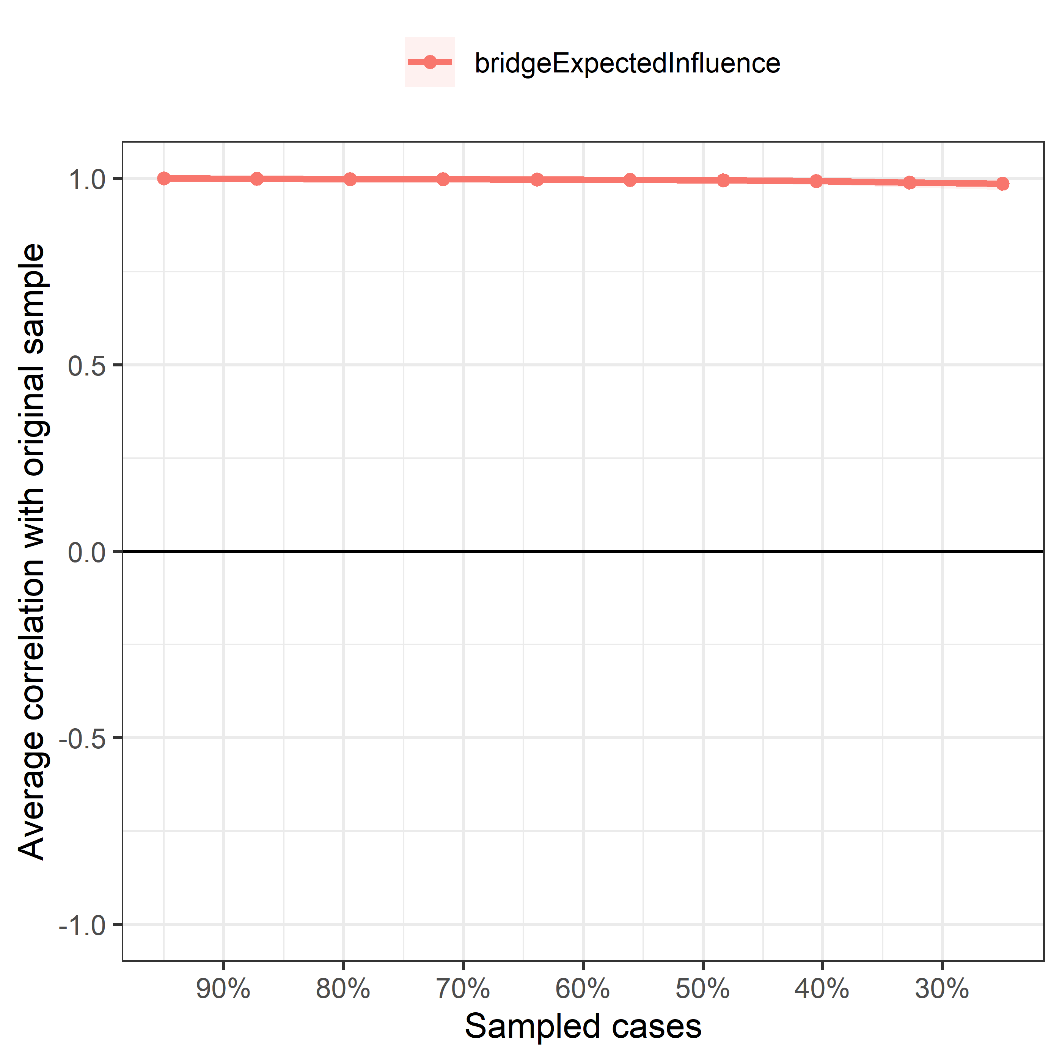
Figure S1. Nonparametric bootstrapped confidence intervals of estimated edges for bridge network. The red line represents the estimated edge, while the dark area indicates the 95% bootstrap confidence interval.
Figure S2. Case-dropping bootstrap test of centrality indices. The x-axis indicates the percentage of cases of the original sample included at each step. The y-axis indicates the correlations between the centrality indices from the original network and the indices from the networks re-estimated after excluding increasing percentages of cases.
